# Delayed Immune Reconstitution Inflammatory Syndrome in an Immunosuppressed Patient With SARS-CoV-2

**DOI:** 10.7759/cureus.19481

**Published:** 2021-11-11

**Authors:** Rafael Garcia-Carretero, Oscar Vazquez-Gomez, Maria Ordoñez-Garcia

**Affiliations:** 1 Department of Internal Medicine, Mostoles University Hospital, Mostoles, ESP; 2 Department of Hematology, Mostoles University Hospital, Mostoles, ESP

**Keywords:** granulocyte colony-stimulating factor, immune reconstitution inflammatory syndrome, immunosuppression, breast cancer, covid-19

## Abstract

Both immune reconstitution inflammatory syndrome (IRIS) and severe coronavirus disease 2019 (COVID-19) are marked by hyperinflammation as a consequence of dysfunction in myeloid cells and increased production of proinflammatory cytokines. Although these features are common to both diseases, their physiopathology remains unclear. Here we report the case of a 63-year-old woman admitted for severe acute respiratory syndrome coronavirus 2 (SARS-CoV-2) infection. In her clinical course, she developed acute respiratory distress syndrome, probably triggered by the use of granulocyte colony-stimulating factor (G-CSF). We hypothesize that G-CSF unmasked IRIS.

## Introduction

Severe coronavirus disease 2019 (COVID-19), which can be the result of an exaggerated immune response, is accompanied by the release of proinflammatory mediators in what is called a cytokine storm. Acute respiratory distress syndrome in immune reconstitution inflammatory syndrome (IRIS) also results from increased production of cytokines and other proinflammatory mediators. Although the specific mechanisms remain unclear, the failure of myeloid cells to properly perform their immunomodulatory role may be involved in both IRIS and COVID-19. IRIS, which is associated with the initiation of antiretroviral therapy, can unmask tuberculosis or cryptococcosis, among other infections. IRIS can also be related to severe acute respiratory syndrome coronavirus 2 (SARS-CoV-2) infection in immunosuppressed patients [1,2].

## Case presentation

In April 2021, a 63-year-old woman was admitted to our hospital with dry cough, fever, tiredness, and myalgia over the previous 10 days. Her condition had worsened over the past four days. Her husband had tested positive for SARS-CoV-2 a few days earlier, so she had visited her general practitioner for a reverse transcription polymerase chain reaction (RT-PCR) COVID-19 test, which had come up positive 10 days before admission. As mentioned, the patient’s clinical condition had been deteriorating since then. She had been diagnosed with breast cancer in 2017. In December 2020 she had been diagnosed with bone-only disease (thoracic spine metastases from T6 to T12), and she had been on letrozole 2.5 mg daily and ribociclib 600 mg daily ever since. Each cycle of ribociclib consisted of three weeks of treatment and one week off.

On physical examination, her temperature was 38°C, her heart rate was 110 bpm, and her blood pressure was 153/58 mm Hg. Her respiratory rate was 18 breaths/min, with oxygen saturation of 89% in room air and 95% with a standard nasal cannula (2 L/min). On auscultation, heart sounds were normal. There were no abnormal lung sounds (crackles or wheezes), but vesicular sounds were diminished in the inferior lobes bilaterally. Abdominal, limb, and neurological examinations were normal.

**Figure 1:**
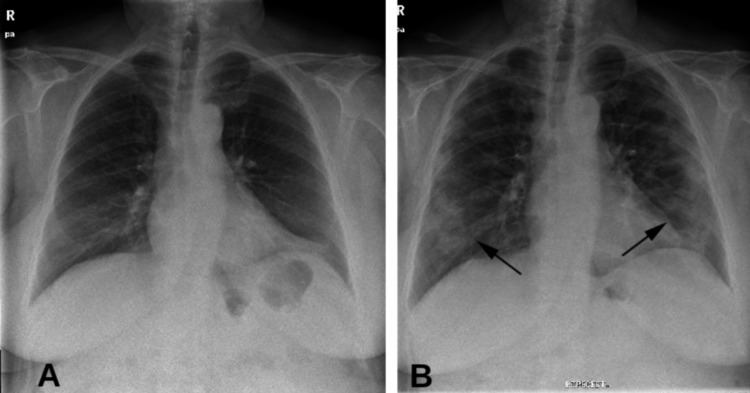
(A) A normal chest X-ray. (B) Peripheral ground-glass opacity (arrows) suggestive of SARS-CoV-2 pneumonia.

Our patient was admitted on day 10 after the onset of her symptoms, and laboratory tests revealed a white blood cell count of 1420 leukocytes/mm3 (60% neutrophils and 29% lymphocytes), hemoglobin of 121 g/L, and a platelet count of 154 × 199/L. C-reactive protein (C-RP) was 15 mg/dL (normal: <5 mg/dL). Arterial blood gas analysis revealed pH 7.43, pO2 63 mm Hg, pCO2 35.7 mm Hg, and bicarbonate 25 mmol/L. Ferritin and D-dimers were normal. Kidney and liver panels were normal, and blood cultures were negative. An RT-PCR test for SARS-CoV-2 was positive, but a chest X-ray showed no abnormalities (Figure 1A). Because the patient presented with respiratory insufficiency, fever, and a positive test for SARS-CoV-2, we hospitalized her. The patient underwent a thoracic computed tomography (CT) scan, which showed no lung abnormalities.

Our first suspected diagnosis was an acute respiratory infection in an immunosuppressed patient due to SARS-CoV-2, without pneumonia, but we could not rule out early-stage pancytopenia/neutropenic fever due to a biologic (ribociclib). We decided to closely monitor the patient’s clinical features, laboratory tests, and chest X-ray. The only treatment we prescribed was pantoprazole, enoxaparin, and paracetamol if she had a fever, but her fever disappeared shortly after admission. On day two postadmission (day 12 since the onset of her symptoms), the patient did not need paracetamol, but she was oxygen dependent (1-2 L/min).

On day six postadmission she had a fever (38.5°C), and blood cultures were obtained (they were negative). Her white blood cell count was 1060 leucocytes/mm3 (350 neutrophils and 650 lymphocytes), and C-RP was 45 mg/dL. D-dimers were normal, but ferritin was 497 mg/dL (normal range: 20-320 mg/dL). Chest X-ray showed no abnormalities, and a thoracic CT scan did not show typical ground-glass opacity. We then established a diagnosis of neutropenic fever, and cefepime and levofloxacin were started. We also began filgrastim (granulocyte colony-stimulating factor [G-CSF]) at a dose of 0.5 million units/kg/day (30 MU in a single subcutaneous injection per day).

On day eight postadmission (day 18 since the onset of COVID-19 symptoms) the patient’s fever disappeared but respiratory insufficiency worsened and oxygen had to be increased to 4 L/min. Leukocyte and neutrophil count recovered to normal ranges, but C-RP increased to 192 mg/dL and chest X-ray showed ground-glass opacity in the mid and lower lobes bilaterally (Figure 1B), which suggested COVID-19 pneumonia.

**Figure 2:**
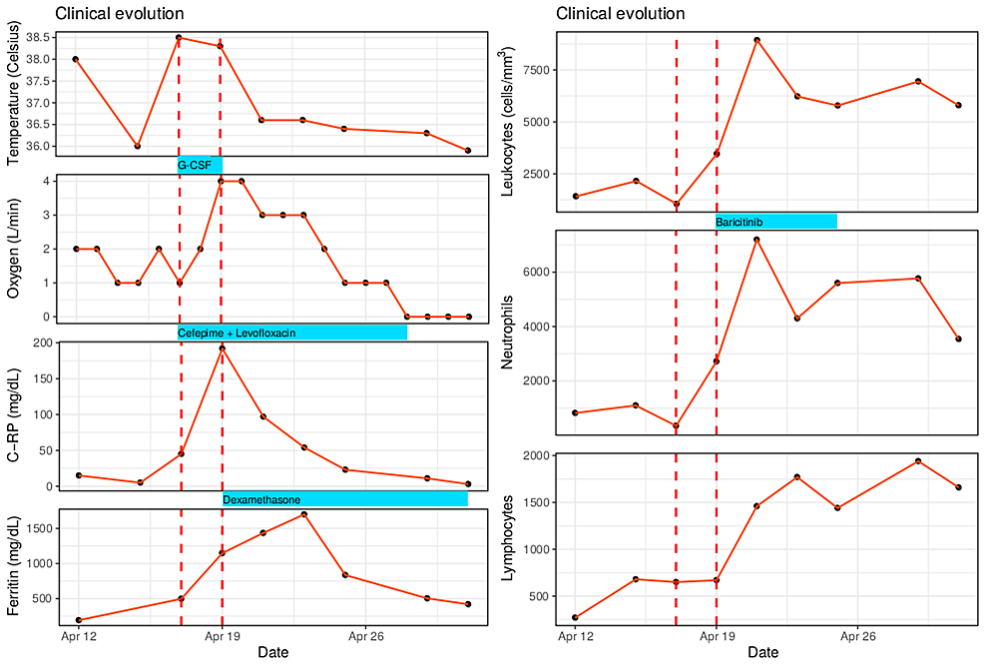
Clinical and laboratory test course of the patient. Temperature curve (in Celsius), oxygen requirement to maintain saturation >95% (in L/min), white blood cell counts, neutrophils, lymphocytes, C‐reactive protein (C-RP), and ferritin.

Dexamethasone 8 mg daily and baricitinib 4 mg daily were started. Our patient recovered over the next seven days. C-RP and ferritin decreased to normal levels, and a chest X-ray at discharge revealed radiological improvement of the pulmonary consolidation. Figure 2 shows the curve of clinical features and laboratory biomarkers.

## Discussion

COVID-19 is often characterized by two stages: a first stage of viral replication with malaise, fever, dry cough, myalgia, or loss of taste and smell, and a second stage of systemic inflammation or cytokine storm [1,2]⁠. The onset of this second stage of hyperinflammation often occurs about 10 days after the onset of COVID-19 symptoms. Given the onset of SARS-CoV-2 symptoms in our patients, pneumonia was expected between days one and four of admission, not on day eight. However, our patient’s condition worsened soon after the first dose of G-CSF. There was a temporal correlation between the first use of filgrastim, the worsening of respiratory insufficiency, the worsening of acute phase reactants (ferritin and C-RP), and the detection of pulmonary ground-glass infiltrates on X-ray. A likely explanation of COVID-19-related IRIS following G-CSF was established.

As discussed previously, we established two main diagnoses: a) mild COVID-19 with respiratory insufficiency but no pneumonia and b) neutropenic fever in a patient on a biologic (ribociclib). The first diagnosis was managed with paracetamol and close monitoring, and the second diagnosis was treated with antibiotics and G-CSF. The interesting aspect of this case report is that we hypothesize that we triggered IRIS with the G-CSF treatment. That is, ribociclib had an immunomodulatory effect on the COVID-19-related hyperinflammation, but G-CSF, apart from improving neutropenia, prompted the COVID-19 pneumonia.

IRIS has been described in patients infected with HIV after the initiation of antiretroviral therapy [3]⁠, often related to preexisting or subclinical infection. Common types of IRIS are tuberculosis, cryptococcosis, or JC polyomavirus-associated IRIS, among others [4]⁠. Regarding COVID-19, although a further explanation is beyond the scope of this paper, the hyperinflammation or cytokine storm seems to be related to myeloid dysfunction and the uncoordinated immune modulation of the T cell response to SARS-CoV-2 [5,6]⁠. HIV-associated IRIS and COVID-19-associated hyperinflammation have some similarities in terms of immune response, although specific mechanisms remain unclear [7]⁠.

Some types of IRIS and severe COVID-19 have been described in patients who received hematopoietic cell transplants during the pre-engraftment period and at the time of immune reconstitution [8]⁠. However, the most similar case report to ours was published by Mertens et al. [9]⁠. Their patient had been diagnosed with nasopharyngeal carcinoma and was on chemotherapy. He was admitted with pancytopenia/neutropenic fever, treated with G-CSF, and developed severe acute respiratory distress syndrome after using G-CSF. In light of the temporal sequence, the authors also hypothesized that G-CSF triggered COVID-19-associated IRIS.

We have several concerns, however. For example, we cannot unequivocally state that our patient developed COVID-19 pneumonia instead of G-CSF-triggered COVID-19-related IRIS. A possible coincidence between the use of G-CSF on the one hand and hyperinflammation and pneumonia due to COVID-19 on the other cannot be excluded. However, our patient’s clinical course was uncommon. Among the general population, the main criteria for hospitalization are often pneumonia and respiratory insufficiency, but our patient was admitted with mild to moderate symptoms (no pneumonia). Instead of developing COVID-19 pneumonia on days 10-14 since the onset of symptoms, the patient developed pneumonia and hyperinflammation on day 18, probably triggered by her first doses of G-CSF. Thus, we hypothesize that our patient’s IRIS was due to the SARS-CoV-2 infection already present at the beginning of G-CSF treatment and thus was not purely coincidental.

Another concern is that SARS-CoV-2 infection can present with leukopenia and thrombopenia, but we did not notice these until day six postadmission (day 16 since the onset of symptoms). Thus, we considered it more likely that these were an adverse effect of ribociclib rather than a consequence of SARS-CoV-2 infection. Our patient’s clinical course was more suggestive of G-CSF-induced IRIS that had unmasked moderate COVID-19 pneumonia than the natural course of COVID-19 pneumonia.

We treated both the IRIS and the COVID-19-induced pneumonia with a corticosteroid because suppressing the inflammatory response can be useful in treating both IRIS and severe COVID-19 [10,11]⁠. The outcome was good, and the patient was discharged on day 25 postadmission.

## Conclusions

Clinicians managing COVID-19 should be mindful of treating patients with SARS-CoV-2 infection with G-CSF, as G-CSF could trigger IRIS. Immunosuppressants or biologics such as ribociclib can have an immunomodulatory effect on the increased activation of myeloid cells but can mask manifestations of severe infection such as COVID-19. If IRIS is suspected in a patient with COVID-19, corticosteroid therapy should be initiated, as it can be highly effective. Physicians should be aware that COVID-19-related manifestations have not been fully described, so treating and reporting IRIS associated with SARS-CoV-2 could be very informative for patients with similar clinical conditions and may help unveil the mechanisms of those exaggerated inflammatory responses.

## References

[1] Ragab D, Salah Eldin H, Taeimah M, Khattab R, Salem R (2020). The COVID-19 cytokine storm; what we know so far. Front Immunol.

[2] Chen J, Qi T, Liu L (2020). Clinical progression of patients with COVID-19 in Shanghai, China. J Infect.

[3] French MA, Lenzo N, John M (2000). Immune restoration disease after the treatment of immunodeficient HIV-infected patients with highly active antiretroviral therapy. HIV Med.

[4] French MA (2012). Immune reconstitution inflammatory syndrome: immune restoration disease 20 years on. Med J Aust.

[5] Merad M, Martin JC (2020). Pathological inflammation in patients with COVID-19: a key role for monocytes and macrophages. Nat Rev Immunol.

[6] Schulte-Schrepping J, Reusch N, Paclik D (2020). Severe COVID-19 is marked by a dysregulated myeloid cell compartment. Cell.

[7] Seddiki N, French M (2021). COVID-19 and HIV-associated immune reconstitution inflammatory syndrome: emergence of pathogen-specific immune responses adding fuel to the fire. Front Immunol.

[8] Malek AE, Adachi JA, Mulanovich VE (2021). Immune reconstitution and severity of COVID-19 among hematopoietic cell transplant recipients. Transpl Infect Dis.

[9] Mertens J, Laghrib Y, Kenyon C (2020). A case of steroid-responsive, COVID-19 immune reconstitution inflammatory syndrome following the use of granulocyte colony-stimulating factor. Open Forum Infect Dis.

[10] Meintjes G, Skolimowska KH, Wilkinson KA (2012). Corticosteroid-modulated immune activation in the tuberculosis immune reconstitution inflammatory syndrome. Am J Respir Crit Care Med.

[11] Horby P, Lim WS, Emberson JR (2021). Dexamethasone in hospitalized patients with Covid-19. N Engl J Med.

